# The Prehistory of Potyviruses: Their Initial Radiation Was during the Dawn of Agriculture

**DOI:** 10.1371/journal.pone.0002523

**Published:** 2008-06-25

**Authors:** Adrian J. Gibbs, Kazusato Ohshima, Matthew J. Phillips, Mark J. Gibbs

**Affiliations:** 1 Yarralumla, Australian Capital Territory (ACT), Australia; 2 Laboratory of Plant Virology, Faculty of Agriculture, Saga University, Saga, Japan; 3 School of Botany and Zoology, Faculty of Science, Australian National University, Canberra, Australia; 4 Curtin, Australian Capital Territory (ACT), Australia; Yale University, United States of America

## Abstract

**Background:**

Potyviruses are found world wide, are spread by probing aphids and cause considerable crop damage. *Potyvirus* is one of the two largest plant virus genera and contains about 15% of all named plant virus species. When and why did the potyviruses become so numerous? Here we answer the first question and discuss the other.

**Methods and Findings:**

We have inferred the phylogenies of the partial coat protein gene sequences of about 50 potyviruses, and studied in detail the phylogenies of some using various methods and evolutionary models. Their phylogenies have been calibrated using historical isolation and outbreak events: the plum pox virus epidemic which swept through Europe in the 20th century, incursions of potyviruses into Australia after agriculture was established by European colonists, the likely transport of cowpea aphid-borne mosaic virus in cowpea seed from Africa to the Americas with the 16th century slave trade and the similar transport of papaya ringspot virus from India to the Americas.

**Conclusions/Significance:**

Our studies indicate that the partial coat protein genes of potyviruses have an evolutionary rate of about 1.15×10^−4^ nucleotide substitutions/site/year, and the initial radiation of the potyviruses occurred only about 6,600 years ago, and hence coincided with the dawn of agriculture. We discuss the ways in which agriculture may have triggered the prehistoric emergence of potyviruses and fostered their speciation.

## Introduction

Two of the 73 genera of plant viruses share equally more than 30% of all recognized species [1]; *Potyvirus* is one of them and *Begomovirus* the other. *Potyvirus* is the largest of six genera in the family *Potyviridae* [1], [2], and is named after its type species, *potato virus Y*. Many potyviruses are damaging crop pathogens. They infect species of most angiosperm taxa in all temperate and tropical climes. They are transmitted by aphids, especially species of *Aphidinae*, when aphids move from plant to plant and probe them in search of their preferred host species, and some are also transmitted in seeds to the progeny of infected plants. Why are there so many potyviruses? Is their dominance recent or ancient? To answer such questions we first need to know when the present day plague of potyviruses originated.

Viruses leave no fossils and their evolutionary rates can only be estimated by linking features of their phylogenies to dates obtained in other ways. A few viruses of animals, notably some orthomyxoviruses and lentiviruses, sometimes evolve so quickly that their evolutionary rates can be estimated by comparing samples collected at different times during an epidemic [3], [4]. Other viruses have phylogenies that are congruent with those of their hosts implying that they have co-evolved [5]–[10], and the co-evolutionary congruence is sometimes complete suggesting that the virus group is as old as its hosts. One genus of plant viruses, the tobamoviruses, shows this sort of relationship [11], [12] suggesting that it first radiated about 100 million years ago, when the asterid and rosid lineages of plants diverged. The taxonomy of potyviruses shows no comparable congruence with host taxonomy, and species from the same lineage may have quite unrelated hosts. For example, the principal hosts of different species of the bean common mosaic lineage are aroids, cucurbits, legumes, orchids and passifloras. The only other published estimates of the rates of evolution of plant viruses are those of wheat streak mosaic tritimovirus (WSMV), which was found to be 1.1×10^−4^ nucleotide substitutions/site/year (ns/s/yr) [13], of rice yellow mottle virus, 4–8×10^−4^ ns/s/yr [14], and of tomato yellow leaf curl begomovirus 4.6×10^−4^ ns/s/yr [15]. These rates are similar to those reported for some populations of viruses of animals [16].

In this paper we report a study of the timescale of potyvirus evolution. The development of methods for inferring and dating phylogenies from gene sequences is currently an active area of research. We have therefore used several different methods, and made limited comparisons between them, to search for a consensus of estimates that will provide a credible timescale for the prehistory of potyviruses.

## Materials and Methods

Potyvirus gene sequences were obtained from the Genbank database using its ENTREZ and BLAST search facilities, and were manipulated using BIOEDIT [17]. The initial collation and alignment of sequences was done using CLUSTALX [18] and its neighbor-joining tree (NJ; [19]) option. The resulting trees were viewed using TREEVIEW [20]. Synonymous and non-synonymous differences between sequences were estimated using the DnDscan method [21] and presented and graphed using EXCEL. Some distance estimates were presented as histograms using SBHistogram (http://www.sb-software.com/sbhisto/)

Only the ‘coherently evolving’ region of the potyvirus coat protein genes (cCP; defined in Results) were used for phylogenetic comparisons; the cCP region of potato virus Y genome (Genbank Reference Sequence NC_001616 [22]) encodes the sequence -DVNAG- at its N-terminus and the C-terminus of the VP at the other. A representative set of 47 different cCP sequences (List S1) was used both as a template for all alignments and as an outgroup together, when required, with that of ryegrass mosaic rymovirus (RGMV) as an outlier to the outgroup. These sequences were aligned via their encoded amino acid sequences using the Transalign program (kindly supplied by Georg Weiller) and CLUSTALX [18] with default parameters, and gave an alignment with 720 nucleotides and gaps (240 codons); the alignment of the cCPs of these ‘outgroup potyviruses’ will be provided on request. The aligned outgroup sequences were checked for incongruent relationships, which might have resulted from recombination, using the RDP package version 3.22 [23] with default settings and a Bonferroni corrected *P*-value cut off of 0.05, and they were also checked by the PHYLPRO program (Weiller 1998).

The phylogenetic relationships of sequences were inferred and compared mostly using the maximum likelihood method PhyML (ML)[24]. Limited tests were also done using the program BEAST [25] to assess whether a Bayesian method gave trees that were of different size or topology from those obtained by ML methods. We also checked whether both of the above methods and also the maximum parsimony method PAUP (version 4.0b10) with the tree branch reconnection [26] and neighbor-joining (NJ) method [19] gave trees with similar topologies. Default settings were used for all analyses except where specified.

To compare the evolutionary rates obtained using different evolutionary models and methods we assembled four small representative sets of aligned cCP sequences. The basis of each set was the 47 outgroup potyvirus cCPs, which provided for each set the same unresolved root (see Results). One set was just the 47 species sequences, to another was added the 29 cCP sequences (List S2) of cowpea aphid-borne mosaic virus (CAbMV), to a third was added 30 representative cCP sequences (List S2) of papaya ringspot virus (PRSV), and the fourth had all three, outgroup plus CAbMV plus PRSV sequences; the four test sets thus contained a total of 47, 76, 77 and 106 sequences, but all had the same root.

For each of the four test sequence sets MODELTEST 3.7 [27] favoured the general time-reversible model [28] with gamma-distributed rate variation [29] and a proportion of invariable sites (GTR+I+G) and the Hasegawa, Kishina, Yano model (HKY+I+G) [30] using both the Akaike information criterion (AIC) and likelihood ratio test [31]. Likelihood scores using a single clock for all branches (strict clock) or branch-specific clocks (relaxed clock) models were obtained in PAUP and compared using the clock likelihood ratio test (http://www.molecularevolution.org/cdc/resources/lrt.php; http://www.stat.tamu.edu/west/applets/chisqdemo.html). Trees were obtained using BEAST with either ‘strict’ or ‘relaxed’ clocks so that the effect of this difference on dating could be assessed; for the ‘relaxed’ clock the rates for different branches were distributed according to a lognormal distribution. Each BEAST analysis involved at least five million Monte Carlo Markov Chain (MCMV) generations with samples taken every 1000, and ESS values assessed by Tracer 1.4 of at least 50; duplicate analyses gave results that differed on average by less than 2% of the mean.

Divergence times were estimated from the full sets of cCP sequences aligned with the outgroup cCPs by comparing pairwise distances inferred as ML estimates by PhyML and date estimates by BEAST using the GTR+I+G model for both. The Newick format trees obtained from PhyML or BEAST were converted to distance matrices using PATRISTIC [32]. The position of a node (single or polytomous) was then calculated as the mean pairwise patristic distance of all pairs of sequences connected through that node; this ‘basal divergence’ is double the mean length of the individual branches, which, combined with an estimate of the date of the node, provide an estimate of their ‘evolutionary rate’.

## Results

### The sequences

There are more than 4,000 potyvirus sequences in the Genbank database. Most are from the 3-terminal region of the genome, which encodes the coat protein gene. The 5′terminal region of the coat protein gene is frequently repetitive, whereas that encoding the core and C-terminus of the coat protein gene is not, and seems to have evolved in a coherent hierarchical way by point mutations and by occasional homologous recombination [33], so we call this region the “coherently-evolving coat protein” (cCP) region and used it for our phylogenetic studies. Trees with closely similar topologies were obtained from all regions of the potyvirid genome, except its variable 5′-terminal region and the 5′-terminal region of the coat protein gene, using maximum likelihood, parsimony or distance methods, and this topology did not change when the genome was divided by windows of fixed length rather than gene by gene [34].

**Figure 1:**
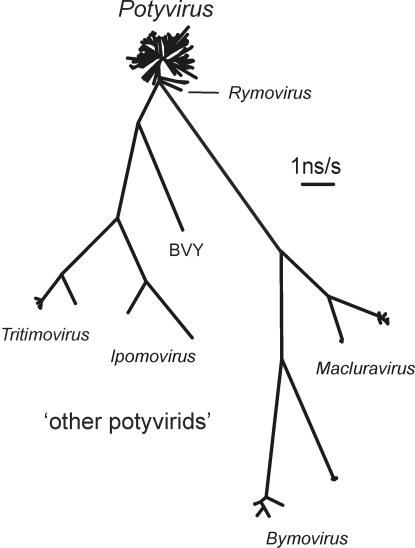
Unrooted phylogenetic tree showing the relationships of the cCP gene sequences of 227 potyvirids including 183 potyviruses. The ‘other potyvirids’ include species of *Macluravirus* (maclura mosaic, *Narcissus* latent and cardamom mosaic viruses), *Bymovirus* (barley mild mosaic, barley yellow mosaic, oat mosaic, wheat spindle streak mosaic and wheat yellow mosaic viruses), *Tritimovirus* (brome streak mosaic, oat necrotic mottle and wheat streak mosaic viruses), *Ipomovirus* (cucumber vein yellowing and sweet potato mild mottle viruses) and *Rymovirus* (ryegrass mosaic, *Agropyron* mosaic and *Hordeum* mosaic viruses). BVY is blackberry virus Y; an unassigned potyvirid species. The tree was calculated from the cCP regions using the PhyML program with the HKY+I+G model.

Phylogenies calculated from the cCP region of 227 sequences representing all genera of the potyvirids place all potyviruses in a compact cluster with a surprisingly uniform radial branch length (Fig. 1 and List S3). The potyvirus cluster was consistently connected in all taxonomies by a short branch to the rymovirus cluster, and the rymoviruses were connected by much longer branches to the other potyvirid genera (Fig. 1).

**Figure 2:**
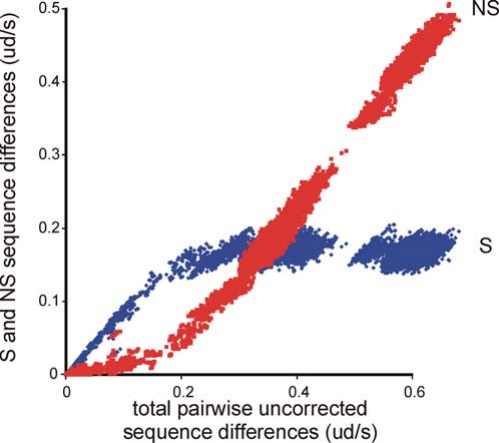
Graph of the number of pairwise synonymous (blue) and non-synonymous (red) differences between the cCP genes of the 227 potyvirids in Fig. 1 plotted against total number of differences. cCP genes of different outgroup potyviruses mostly have total differences of around 0.36 uncorrected differences/site, corresponding to 64% identity or 1.5 nucleotide substitutions/site.

There are several ways in which the distinctive topology of the potyvirus cluster may have arisen. Most likely it originated as a major speciation event, a star-burst, from which all lineages have diverged at similar rates with several of those lineages also forming small sub-lineages. Alternatively, if potyvirus genes have a limited capacity for change, then the star-burst may reflect mutational saturation. However when the pairwise differences of the 227 potyvirid cCP sequences were separated into non-synonymous (NS) and synonymous (S) differences and were plotted against the total number of differences (Fig. 2), S differences can be seen to have saturated and not increase further after total differences exceeded about 0.20–0.30 uncorrected differences/site (ud/s), whereas NS differences between the cCPs of potyvirus species had not saturated. The large number of points around 0.36 ud/s represent pairwise comparisons between potyvirus species, whereas comparisons between the cCPs of species of different potyvirid genera ranged from 0.5–0.7 ud/s. This indicates that, unless the mutability of potyvirus cCP genes is quite different from that of the cCPs of other potyvirids, then the potyvirus topology is not the consequence of mutational saturation. Finally there is also the possibility that the potyvirus topology is the result of extensive recombination between sequences. However a check of the 47 outgroup potyvirus cCPs using the RDP3 package of programs found only five cCPs with tentative evidence of recombination, and that was given by only with a single method, Siscan, out of the seven in RDP3 and by PHYLPRO, and only one cCP, that of onion yellow dwarf virus, gave tentative evidence of recombination by two methods. So we conclude that the topology of the potyviruses is most probably a star-burst resulting from a major radiation event.

### Dating the Potyvirus Major Radiation

Only in the last decade have significant numbers of potyvirus gene sequences been determined, and there is no reason to believe that comparisons of non-contemporaneous sequences obtained over such a short period of time would allow the ‘time signal’ to be distinguished with any accuracy from the ‘population variation signal’, as even the simplest calculation using uncorrected distances shows that it would involve a several hundreds-fold extrapolation. Therefore we searched for historical isolation and outbreak events that correlated with the phylogenetic relationships of potyvirus cCP sequences, and could provide longer time scales from which to estimate the date of the radiation. Four independent lines of evidence were found:

#### Plum pox virus

**Figure 3:**
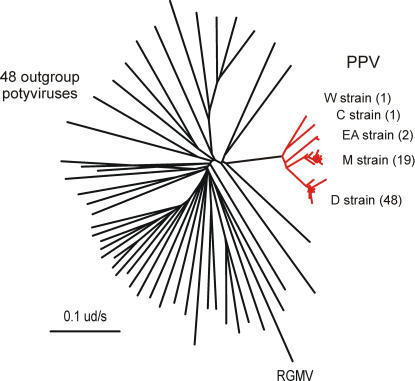
Radial neighbor-joining tree of the cCP genes of 71 isolates of plum pox virus (red branches) and 47 outgroup potyviruses and ryegrass mosaic rymovirus (black branches). The number of isolates of each strain cluster is in parentheses.

Long historical records exist for most *Prunus* species (stonefruits). Chinese literature records cultivation of the peach, *Prunus persica*, over the past 3000 years. Pliny (23–79AD) mentioned twelve distinct varieties of plum, some of which were taken to Great Britain by the Romans, where they were recorded in William Langland's *Piers Ploughman* (1362) [35]. Significantly however, there is no known record of the very obvious and damaging symptoms of plum pox virus (PPV) until around 1915 in Bulgaria. Plum pox, “Sharka” in Slavic, then spread north and east across Europe in a well-documented epidemic infecting most cultivated and wild *Prunus* species [36], and it has recently spread to the Americas; it was first found in Chile in 1992 and in North America in 1999 [37]. Comparisons of the 67 cCP genes (List S4) of PPV isolates collected from throughout the area of this outbreak (Fig. 3) placed most of the isolates into two major clusters. These two clusters had been distinguished by serological tests before gene sequencing became routine, and they are called D (Dideron) and M (Marcus) strains of PPV. The D strain cCP sequences came from isolates collected throughout Europe, Eurasia and the Americas, whereas the M strain cCPs came only from Europe, and there are also four cCP sequences from three other distinct strains. The mean difference of all pairs of sequences linked through the basal node of the M strain cluster was 0.028+/−0.004 nucleotide substitutions/site (ns/s), but only 0.009+/−0.002 ns/s for the D strain. It might seem most likely that the epidemic of the last 90 years was of the more widely dispersed D strain. However the M strain causes much more severe symptoms and “is considered to be the epidemic form of the virus” [36], whereas the D strain may have spread further, but more recently, merely because infected plants show few or ephemeral symptoms and so plants chosen for propagation and dispersal may have been infected with this strain until more sensitive detection methods stopped its inadvertent spread in horticultural stock. Plum pox disease first appeared in Bulgaria 90 years ago, but the epidemic in stonefruits probably started several years before. If we assume therefore that the diversity of the PPV-M population has been acquired over the past 90 years plus an ‘eclipse’ period of 10 years during which the significance of the disease was not recognized or recorded, then the evolutionary rate of PPV-M will have been 1.4×10^−4^ ns/s/yr. The PPV cCP sequences differ from those of the outgroup potyviruses by a mean of 1.269+/−0.0696 ns/s, so that if the expected differences are linearly related to elapsed time, then the potyvirus radiation possibly occurred around 4532 years before present (YBP).

#### Australian potyviruses

Agriculture was not established in Australia before the continent was colonized by Europeans in the late 18^th^ century [38]. Thirty eight potyvirus species have been isolated in Australia and had their cCP sequences determined [39](List S5). Of those thirty eight viruses, twenty are cosmopolitan (Table 1) in that they are also found elsewhere in the world, whereas eighteen (Table 2) have only been found in Australia. Most of the cosmopolitan viruses are well studied crop pathogens that cause significant damage wherever they occur. Most of the Australian populations of the cosmopolitan viruses are much less diverse than their corresponding world populations, none are more diverse and some have only recently appeared in Australia. They are most probably immigrants carried to Australia during the past two centuries. By contrast the eighteen endemic potyviruses are mostly found in native plants or weeds, only two infect crop species, and most of their populations are much more diverse than those of the immigrant viruses indicating that they have been in Australia longer.

**Table 1:** Potyviruses found both in Australia and elsewhere.

Name	Number of Australian sequences	Divergence^1^ of Australian sequences	Divergence from closest overseas sequence	Divergence of world population	Divergence from outgroup potyviruses^2^
*Apium* virus Y	3	0.027	NA^3^	-	1.467
Celery mosaic virus	4	0.003	0.020	0.020	1.458
Johnson grass mosaic virus	25	0.034	0.133	0.133	1.645
Papaya ringspot virus	6	0.015	0.025	0.186^4^(0.378)	1.334(1.329)
Sugarcane mosaic virus	12	0.034	0.026	0.316	1.414
Sweet potato feathery mottle virus - strain C	5	0.009	0.028	0.047	1.595
Sweet potato virus Y	5	0.003	0.012	0.189	1.520
Mean	8.6	0.018	0.041	0.146	1.490

1 Divergences calculated from ML trees (GTR+I+G) of the cCP sequences as the mean pairwise nucleotide substitutions/site of sequences linked through the basal node.

2 Outgroup potyviruses of the same lineage omitted.

3 NA, not applicable; a partial cCP sequence of a New Zealand isolate has been reported, its sequence is 97% identical to the Australian sequences.

4 The atypical cCP sequence of PRSV from *Lagenaria siceraria* in Pakistan (AB127935) was excluded; divergence when that sequence is included is given in parentheses.

Note: cCP sequences have also been reported for Australian isolates of bean common mosaic, bean yellow mosaic, clover yellow vein, *Ornithogalum* mosaic, pea seed-borne mosaic, peanut mottle, *Pleione* virus Y, sweet potato feathery mottle-strain RC, turnip mosaic and watermelon mosaic viruses, but their phylogenies indicate that different lineages have entered Australia, and hence on more than one occasion. Partial cCP sequences also available for *Euphorbia* ringspot virus (1 sequence), potato virus Y (3 sequences) and zucchini yellow mosaic virus (4 sequences) with nearest overseas sequences with 98%, 97–99% and 93–100% identities respectively in BLAST searches.

**Table 2:** Potyviruses found only in Australia.

Name	Number of Australian sequences	Divergence^1^ of Australian sequences	Nearest viral sequence	Maximum identity^2^
Carrot virus Y^3^	3	0.007	Celery mosaic virus	81%
Ceratobium mosaic virus	5	0.109	*Tricyrtis* virus Y	77%
*Hardenbergia* mosaic virus	30	0.297	Passionfruit woodiness virus-WA	80%
Passionfruit woodiness virus-NSW	3	0.037	Siratro 1 virus Y	85%
Passionfruit woodiness virus-WA	5	0.049	*Clitoria* virus Y	84%
*Pterostylis* virus Y	2	0.011	*Ornithogalum* mosaic virus	90%
mean	8	0.085	-	82.8%

1 divergences calculated from ML trees (GTR+I+G) of the cCP sequences as the mean pairwise nucleotide substitutions/site of sequences linked through the basal node.

2 Maximum identity in BLAST searches of the Genbank database.

3 Carrot virus Y infects carrot crops throughout southern Australia, and has all the features of a recent immigrant rather than an endemic.

Note: Single cCP sequences are also known for *Clitoria* virus Y, *Diuris* virus Y, *Eustrephus* virus Y, *Hibbertia* virus Y, *Kennedya* virus Y, *Passiflora foetida* virusY, *Rhopalanthe* virus Y, *Sarcochilus* virus Y, Siratro 1 virus Y and Siratro 2 virus Y. The nearest sequences to these had 84%, 78%, 83%, 82%, 74%, 89%, 80%, 83%, 86% and 84% maximum identities respectively in BLAST searches of the Genbank database. Single partial cCP sequences also available for *Dianella* chlorotic mottle virus and *Glycine* virus Y and the nearest sequences to these had 82% and 84% identities respectively in BLAST searches of the Genbank database.

There is no information about when individual immigrant viruses entered Australia, so it is not possible to compare the population diversity of each virus with its date of entry and thereby directly obtain measures of their evolutionary rates. However, if our conclusion that they have all entered during the past two centuries, is correct, then the average diversity of the populations can be assumed to have accumulated while they have been in Australia.

Phylogenies calculated from the cCP sequences of the immigrant viruses indicated that seven probably entered Australia on only one occasion. The populations of these viruses had a mean basal divergence of 0.0176 ns/s (Table 1), and on average differed from the nearest overseas cCP by only twice as much, 0.0327 ns/s. The cCP sequences of the Australian immigrant viruses differed from those of the outgroup potyviruses by a mean of 1.4904 ns/s. The diversity and number of plants imported to Australia has greatly increased since agriculture was established two centuries ago, especially since the Second World War when the number of vessels, tonnage of goods and number of human migrants has increased exponentially [40] so it is likely that the mean time of potyvirus entry is less than 100 years. If we assume it is 75 years then the average evolutionary rate of the potyvirus populations is 1.173×10^−4^ ns/s/yr and hence the potyvirus radiation occurred 6353 YBP.

#### Maritime trade and the great plant diaspora

**Figure 4:**
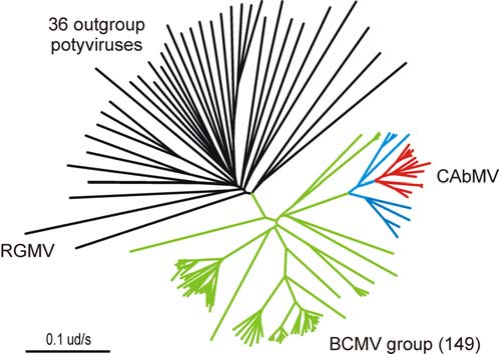
Radial neighbor-joining tree of the cCP genes of 149 isolates of different species of the bean common mosaic virus (BCMV) group (green branches), with 36 outgroup potyviruses and ryegrass mosaic rymovirus (RGMV) (black branches) as an outgroup; the other 11 ‘outgroup potyviruses’ were of the BCMV group. The BCMV group isolates included 24 isolates of cowpea aphid-borne mosaic virus, eight of which came from Africa (blue branches) and 16 of which came from South America (red branches); it also included single isolates of 11 species in the outgroup potyviruses.

**Figure 5:**
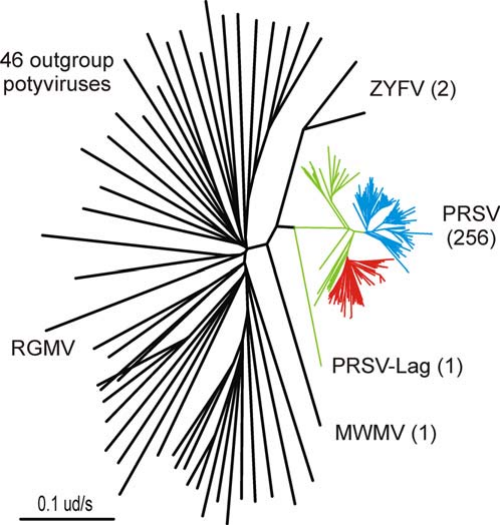
Radial neighbor-joining tree of the cCP genes of 256 isolates of papaya ringspot virus (coloured branches), two of zucchini yellow fleck virus (ZYFV), one of Moroccan watermelon mosaic virus, with 46 outgroup potyviruses and ryegrass mosaic rymovirus (RGMV) (black branches) as an outgroup. The PRSV isolates came from the Indian subcontinent (green branches), or from East Asia (blue branches) or from the Americas (red branches). The number of isolates of each species is in parentheses.

The discovery of America by Columbus in 1492 is traditionally taken to be the event that initiated long distance maritime trade. This linked all parts of the world and over the following two centuries dispersed most cultivated plants from the restricted regions where they had been independently domesticated and grown during the previous 10 to 13 millenia [41]–[43]. Of the quarter million or so species of flowering plants, only about 1,800 of them have been “brought into cultivation” [44], and “only about 100 yielded valuable domesticates” [43]. More than five centuries ago only the calabash gourd, *Lagenaria siceraria*, and the coconut, *Cocos nucifera*, were present in both the Old and New Worlds, and the sweet potato or kumara, *Ipomoea batatas*, was grown in both the Americas and Polynesia [44]. Maritime trade carried most of the others to all corners of the world, and viruses that could travel with them probably did. We therefore searched phylogenies of potyviruses present in both the Old and New Worlds to find those with sub-lineages found only in either the New or Old World. We presume that such disjunct populations were probably transported between the Old and New Worlds and are at most around 500 years old. So far we have examined phylogenies of more than 50 species and found two of this sort, namely those of cowpea aphid-borne mosaic (CAbMV) (Fig. 4) and papaya ringspot (PRSV) viruses (Fig. 5).

CAbMV infects various species and cultivars of cowpea, and also many other species, and is transmitted to a large proportion of the seed of infected cowpea plants. Cowpeas were first domesticated in West Africa [45], and were taken from there to south and east Asia [46], where their name, *catjang*, is from the ancient Indian language Sanskrit and hence at least 3,000 years old. They were also grown by the Romans and Greeks. Several published CAbMV cCP sequences are from African isolates and one from passion fruit with woodiness disease in South Africa [47], but the majority are from South American isolates from passion fruit and a few from *Arachis hypogaea* and *Crotalaria* spp. [48]. A phylogeny of the 25 reported CAbMV cCP sequences (Fig. 4 and List S6) shows that the ten isolates from Africa are the most diverse and closest to the potyvirus radiation, whereas the 15 from South America form a monophyletic subcluster within the CAbMV tree; two South American sequences (DQ397525/6) are recombinants with sequence regions from an unknown potyvirus and were not included in the analysis, and one isolate found in plants grown in the USA from African seed [49] clusters with African isolates. The basal node of the 16 South American cCP sequences linked sequences that differed by a mean of 0.1397+/−0.024 ns/s. CAbMV is a member of the bean common mosaic lineage of potyviruses, so all ten other members of that lineage were excluded from the PhyML analysis that showed that the 16 South American CAbMV cCPs differed from the outgroup potyviruses by a mean of 1.555+/−0.143 ns/s. Travel between Africa and the Americas started around 1500 AD; Brazil was discovered inadvertently in 1502 and slaves taken there from Africa soon afterwards [50], [51]. It is very likely that seeds of cowpea, a staple food of tropical Africans, were taken too [35], [52]. So if we assume that the South American CAbMV lineage was established by infected seed carried by slaves from Africa 500 YBP then the evolutionary rate of CAbMV is 1.397×10^−4^ ns/s/yr, and the potyvirus radiation was, at most, 5565 YBP.

The other virus that was probably involved in the great plant diaspora is papaya ringspot virus (PRSV). This virus causes a very damaging disease of papaya and is only distinguished from a potyvirus of cucurbits, originally called watermelon mosaic virus 1 [53], by its ability to infect papaya. The two viruses are now known as the papaya and watermelon strains of PRSV, namely PRSV-P and PSRV-W, but gene sequence evidence shows that individual PRSV-P populations have arisen independently from local PRSV-W populations in regions where papaya has been introduced as a crop [54], [55]. The cCP sequences of over 300 isolates of PRSV have been reported and form a complex phylogenetic tree (Fig. 5 and List S7). The most diverse are those from India and Sri Lanka, and, excluding one atypical Pakistan isolate from the calabash gourd (AB127935) that should perhaps be considered a distinct species, the world population of PRSV has a basal node divergence of 0.186+/−0.019. Two major PRSV radiations have arisen from different branches of the Indian population. The earliest includes all the isolates in east and south east Asia (China, Indonesia, Malaysia, Philippines, Taiwan, Thailand and Vietnam). The other includes all the isolates from the Americas with small separate sub-clusters of isolates from Hawaii, Taiwan and Australia, and has a basal node divergence of 0.075+/−0.012 ns/s. The mean difference between the American lineage PRSV cCPs and the outgroup potyviruses is 1.494+/−0.148 ns/s. Thus, if the evolutionary rates of PRSV and CAbMV are similar then the basal node divergences of their American populations, 0.075 and 0.1397, suggest that PRSV was carried to the Americas only 300YBP. This difference in the basal node divergences of the two populations was confirmed in all the tests done with different evolutionary models described below (Tables 3 and 4).

**Table 3:** Results of PhyML analyses with test sequence data.

Model used	Sequences^1^	Potyvirus divergence; mid-tree^2^	Potyvirus divergence; outgroup to CAb or PRS^3^	Species divergence^4^	
GTR+I+G	Outgroup	1.460	NA^5^	NA	a
	OG+CAb	1.666	1.742	0.153	b
	OG+PRS	1.525	1.447	0.074	c
	OG+CAb+PRS−CAb	1.669	1.664	0.276	d
	OG+CAb+PRS−PRS	1.669	1.483	0.171	e
	Mean of rows abcd	1.580			
	Mean of rows bcd	1.620			
	Mean of rows bcde		1.584		
HKY+I+G	Outgroup	1.330	NA	NA	a
	OG+CAb	1.508	1.542	0.149	b
	OG+PRS	1.372	1.341	0.072	c
	OG+CAb+PRS−CAb	1.550	1.592	0.222	d
	OG+CAb+PRS−PRS	1.550	1.450	0.172	e
	Mean of rows abcd	1.440			
	Mean of rows bcd	1.477			
	Mean of rows bcde		1.481		

1 See Methods and Supporting Information Lists S1 and S2. OG, outgroup; CAb, CAbMV cCP sequences; PRS, PRSV cCP sequences.

2 Mid-tree divergence (nucleotide substitutions/site). The mean pairwise difference of cCP sequences linked through the midpoint of the tree.

3 Outgroup divergence (nucleotide substitutions/site). The mean pairwise difference between the outgroup cCP sequences and every American CAbMV or PRSV cCP sequence.

4 Species divergence (nucleotide substitutions/site). The mean pairwise difference of sequences linked through the basal nodes of the clusters of the American CAbMV (rows b and d) or the PRSV (rows c and e) cCP sequences.

5 NA, not applicable.

**Table 4:** Results of BEAST analyses of test sequence data.

Model used	Sequences^1^	Root age^2^	Mid tree age^3^	Species age^4^	
GTR+I+G	Outgroup	22392.7	35364	NA^5^	a
strict clock	OG+CAb	8404.5	8094.9	653.9	b
	OG+PRS	8356.9	8484.4	290.2	c
	OG+CAb+PRS-CAb	7932.3	8609.4	633.4	d
	OG+CAb+PRS-CAb			292.5	e
	mean of rows abcd	11771.6	15138.21		
	mean of rows bcd	8231.2	8396.3		
	mean of rows bcde			467.5	
GTR+I+G	Outgroup	27167	34967.8	NA	a
relaxed clock	OG+CAb	9357	8944.3	687.9	b
	OG+PRS	8527.7	8295.7	700.2	c
	OG+CAb+PRS-CAb	8630.5	10368.12	598.3	d
	OG+CAb+PRS-CAb			266.8	e
	mean of rows abcd	13420.5	15644		
	mean of rows bcd	8838.4	9202.7		
	mean of rows bcde			563.3	
HKY+I+G	Outgroup	12603.8	14697.5	NA	a
strict clock	OG+CAb	7746.5	7013.5	472.9	b
	OG+PRS	7570.4	6677.4	253.1	c
	OG+CAb+PRS-CAb	7120.6	6643.04	459.3	d
	OG+CAb+PRS-CAb			255	e
	mean of rows abcd	8760.3	8757.9		
	mean of rows bcd	7479.2	6778		
	mean of rows bcde			360.1	
HKY+I+G	Outgroup	12780.9	11170.3	NA	a
relaxed clock	OG+CAb	8776.3	8996.2	653.6	b
	OG+PRS	7736.9	7447.1	645.9	c
	OG+CAb+PRS-CAb	7736.1	6276.2	474.9	d
	OG+CAb+PRS-CAb			254.9	e
	mean of rows abcd	9257.6	8472.4		
	mean of rows bcd	8083.1	7573.2		
	mean of rows bcde			507.3	

1 See Methods and Supporting Information: Lists S1 and S2. OG, outgroup, CAb, CAbMV cCP sequences. PRS, PRSV cCP sequences.

2 Root age. The mean tree root height as ‘years before present’ (YBP) calculated by BEAST given a prior substitution rate of 1.2×10^−4^ substitutions/site/year.

3 Mid-tree age. The mean divergence (YBP) of all pairs of cCP sequences linked through the mid point of the tree estimated from the two most distant sequences.

4 Species age. The mean divergence (YBP) of the American CAbMV (rows b and d) or PRSV (rows c and e) cCP sequences linked through the basal node of each cluster.

5 NA, Not applicable.

There is, however, no published experimental evidence that PRSV is seed-borne, but the fact that its phylogeny indicates that it has migrated, at different times, from India via East Asia to several Pacific islands and, separately, from India to the Americas and from there, recently, to two Pacific islands and Australia is only plausibly explained by seed transmission. Published results of tests found no seed transmission of PRSV in papaya and *Cucurbita* spp. [56], but these are both natives of the Americas, and are probably recent hosts, whereas the phylogeny of PRSV indicates that its long term hosts are Old World cucurbits, and it is therefore much more likely to have been carried in seeds of plants, such as *Citrullus lanatus*, *Cucumis* spp., *Lagenaria siceraria*, *Luffa* spp., *Momordica charantia* and *Trichosanthes cucumerina* [52].

The earliest trade between India and the Americas was established at least 50 years later than that between Africa and South America. Goa was annexed in 1510 by the Portugese and became the base for repairing and provisioning their Asian fleet, which traded spices and silks to Europe from outposts such as Macau. It is possible that PRSV may have been carried in infected seed directly from Goa to Bahia (now called Salvador) in the late 16^th^ century, however very few boats made that trip [57], and it is more likely to have been carried to South America via Europe probably in the early 18^th^ century. At that time the Europeans, especially the British and the French, as part of their imperial ambitions, collected and distributed plants through the worldwide networks of botanic gardens they had established [58]–[60]; “Kew Gardens under (Joseph) Banks direction assumed the task of acting as the British Empire's botanical clearing house” [61], and its 1768 list of plants [62] includes 21 cucurbit species including all but one of those named above. So, although PRSV might have been transported from India to South America as early as 450 YBP, it is more likely to have been carried there only 300 years ago. If the more recent date is correct then the evolutionary rate of PRSV is 1.25×10^−4^ ns/s/yr and the major radiation of the potviruses 5976 YBP.

### Dating by other models and methods

Quantitative comparisons were also made of trees calculated from four representative sets of aligned cCP sequences. These were comprised of the outgroup potyvirus cCPs aligned with either 29 CAbMV or 30 PRSV cCPs, or both (see Methods). Trees were obtained using PhyML [24] and BEAST [25]. Comparisons of strict and relaxed clock models by clock likelihood ratio tests of the sequences favoured the relaxed clock (branch-specific rates) model, and the BEAST analyses of the four representative sets confirmed this as, with the relaxed clock, all sets of sequences gave only positive ‘coefficient of variation’ values; mean 0.245 and mean lower and upper 95% highest probability density values of 0.145 and 0.335.

The PhyML tests (Table 3) showed that estimates of the age of the potyvirus radiation obtained using the HKY+I+G model were, on average, about 10% less than those obtained with the GTR+I+G model. The only other consistent difference between the two models was that the root of the tree was apparently about 10% less distant when it was estimated from the outgroup sequences alone (i.e. when all sequences were from different species), than when one or two of the species was represented by several sequences. There was no consistent difference in the apparent age of the root of the tree when that position was calculated as the midpoint of all the sequences in the tree rather than the mean pairwise difference between the outgroup sequences and the species represented by several sequences (i.e. CAbMV or PRSV).

However the PhyML tests also showed that the apparent positions of the basal nodes of the CAbMV and PRSV clusters were clearly influenced by what other sequences were present in the dataset (Table 3). For example the node of the South American CAbMV sequences was at 0.153 ns/s when calculated from the ‘CAbMV plus outgroup’ sequences, but when the PRSV sequences were also added the node of the South American CAbMV sequences was estimated as 0.276 ns/s. This will affect attempts to estimate the age of the radiation for if it is assumed that CAbMV first entered South America 500 YBP, then the age of the potyvirus radiation estimated from the ‘CAbMV plus outgroup’ tree is 5444 YBP, whereas it is only 3023 YBP from the ‘CAbMV plus PRSV plus outgroup’ tree. Therefore for our dating analyses we used, where possible, test sequences from just a single species with the outgroup sequences.

Most, but not all, of the results obtained in the BEAST tests (Table 4) were similar to those obtained with PhyML. For example both methods found the age of the potyvirus radiation given by the HKY+I+G model to be less than that given by the GTR+I+G model by a similar amount and, importantly, the BEAST tests showed that the ages of nodes estimated using the ‘uncorrelated lognormal (LN) relaxed clock’ were around 10% older than those obtained using a ‘strict clock’. The two methods used to estimate the position of the potyvirus radiation from the trees gave indistinguishable results. The greatest difference between PhyML and BEAST results was when the radiation was estimated from single representative sequences of each species, rather than when the dataset included sequences also providing intra-species variation. The ML estimate of the radiation was then about 10% less distant, whereas the BEAST estimate was 1.7 to 2.7 times more distant.

In summary, the most consistent date estimates obtained by either PhyML or BEAST were those obtained with datasets that contained a mixture of inter-species and intra-species. These estimates were similarly variable whichever method was used; the standard deviations were from 3% to 12% of the means. The HKY+I+G model gave node estimates about 10% less than the GTR+I+G model, and the LN relaxed clock gave estimates about 10% more than a strict clock. The outgroup dataset alone gave inconsistent results. The CAbMV and PRSV sequences used for these tests were representative of those used in the main dating analyses described above, so it is of interest that all the results (Tables 3 and 4) showed the South American CAbMV lineage to be about twice as divergent as the American PRSV lineage, and hence possibly twice as old.

## Discussion

Four independent lines of evidence have given surprisingly similar ML estimates (Table 5) of the date of the initial major radiation of potyviruses. They range from 4532 YBP to 6353 YBP and are based on independent historical isolation and outbreak events over the past 500 years. Table 5 also shows the large effect that the ‘extrapolation ratio’ has on the possible accuracy of the estimates; when the estimate is based on a branch node dated as 75 YBP then an error of one decade in that dating alters the estimated radiation time of the potyviruses by 847 years, whereas when the node is dated as 500 YBP then a 10 year error changes the radiation time by only 111 years.

**Table 5:** Estimates of the date of the major radiation of potyviruses.

Virus^1^	Rate of evolution (×10^−4^ ns/s/yr)	Length of branch from radiation (ns/s)	Estimate^2^ of radiation date (YBP)	Calibration node date^3^ (YBP)	Accuracy^4^: radiation years per node decade.
PPV	1.40	0.6354	4532	100	453
Australian potyviruses	1.173	0.7452	6353	75	847
American CAbMVs	1.397	0.778	5565	500	111
American PRSVs	1.25	0.747	5976	300	198
Mean of viruses #2-4	1.273	0.7567	5965	-	-

1 See text for name abbreviations and descriptions of viruses.

2 See text for details of estimates.

3 The date (YBP) of the node used to calibrate each evolutionary rate estimate.

4 The number of years that the estimated date of the major radiation would change for every decade that the assumed date of the node is changed.

We have most confidence in the results produced by the Australian potyviruses and the American CAbMV and PRSV populations. The mean potyvirus radiation date from these is 5965+/−394 YBP and the mean evolutionary rate is 1.273×10^−4^ ns/s/yr. The large possible extrapolation inaccuracy of the Australian potyvirus evidence is offset by the fact that the data comes from several viruses

We have least confidence in the results provided by the PPV epidemic data, as two different strains of the virus with seemingly different evolutionary rates were involved in the epidemic. Also, the time between the start of the outbreak and when it was first recorded is unknown. This ‘eclipse phase’ may have been more than the decade that we assumed, especially as, at the time, plant pathology was in its infancy. Indeed if the calculation is reversed, using the average of the three other radiation estimates, 5965 YBP, then we find that the PPV epidemic probably started about four decades before it was noticed, and this is entirely possible.

Thus, to summarize, the ML analyses using a ‘strict clock’ and relying on the data obtained from the Australian and American potyvirus incursions indicate that the evolutionary rate of potyviruses is around 1.273×10^−4^ ns/s/yr and their major radiation occurred 5965 YBP. However analyses of the data indicate that a relaxed clock would be more appropriate than a strict clock, and comparisons of representative test cCP sequences have shown that a relaxed clock analysis finds an evolutionary rate 10% less, and hence a date for the potyvirus radiation 10% greater, than a strict clock analysis. Thus our studies indicate that the potyvirus cCP region most probably evolves at a rate of 1.15×10^−4^ ns/s/yr and the potyvirus major radiation occurred 6,560 YBP.

The evolutionary rate we have estimated for potyviruses is similar to that of 1.1×10^−4^ ns/s/yr of the mite-borne potyvirid, wheat streak mosaic tritimovirus (WSMV) [13], which was first found in American wheat crops in the 1920s [63] having arrived from Europe shortly before. Other similar published evolutionary rates of plant viruses are those for rice yellow mottle virus, which was estimated from samples collected over 40 years to be 4–8×10^−4^ ns/s/yr [14], and of tomato yellow leaf curl begomovirus estimated to be evolving at 4.6×10^−4^ ns/s/yr from samples collected over 18 years [15]. Although these estimates are for different viruses, and so the differences may be species specific, it is noticeable that the evolutionary rates based on samples collected over a short time span (18 and 40 years) are several-fold greater than those based on longer time scales (100 years or more), as has been noted in studies of the rates of evolution of cellular organisms [64], [65].

Not all plant viruses evolve so quickly. The tobamoviruses show evidence of having co-evolved with their hosts, and so it is possible they are more than 100 million years old [11], [12] and evolving 10,000 times more slowly than potyviruses. However this range of evolutionary rates is not exceptional as viruses of animals cover the same range, indeed it is possible that a single ‘virus’ may cover that range by changing its ‘life style’; the *pol* gene of an endogenous lentivirus of European rabbit has been found to have homologues in exogenous (i.e. infectious) lentiviruses [66].

Our results place the potyvirus radiation in the centre of the current geological epoch, the Holocene, during which agriculture and complex human civilization has developed; and even if there had been fortuitously reinforcing combinations of errors in our methods they would have been unlikely to place our estimate of the radiation date outside the Holocene. Is this merely a co-incidence, or was agriculture itself responsible for potyvirus emergence and subsequent speciation? Modern humans first appeared during the Pleistocene around 250,000 years ago, but there is no evidence that animals and plants were domesticated until the last 8,500 to 13,000 years. Agriculture started in at least nine independent regions of the world, each with a different suite of animals and plants [43]. This suggests that the invention of agriculture was driven by a global trigger, rather than by local conditions. The Pleistocene was a period of greatly fluctuating climate. By contrast the Holocene, which started about 11,500 years ago, has been a period of unusually stable and predictable climate [67], [68]. Richerson and colleagues [69] have convincingly argued that the Pleistocene climate was too changeable to allow the domestication of animals and plants. However the predictable climate in the Holocene drove the development of agriculture. It allowed human ‘hunter gatherers’ to increase in numbers and over-exploit the resources that had supported them in the Pleistocene. In seeking new resources to support themselves, humans progressively selected and domesticated species that provided food and other materials. Successive rounds of selection and dependence provided the ‘selective ratchet’ leading to domestication of suitable species, but this was only possible because the predictable climate allowed the lengthy selective process of adaptation and domestication to proceed. Thus agriculture appeared more or less contemporaneously, but independently, in nine regions of the world linked only by the climatic predictability and stability of the Holocene.

The ‘emergence’ and spread of viruses also depends crucially on climate [70], [71]. We therefore suggest that the stable Holocene climate also fostered viruses with particular ecological life styles that preadapted them to a world increasingly dominated by agriculture. Potyviruses are spread by migrating aphids. More than 200 species of aphids spread potyviruses [2], [72], and most are from the subfamily Aphidinae (*Myzus*, *Macrosiphum* and *Myzus* species) [73]; each potyvirus may be spread by many different aphid species and each aphid species may transmit many potyviruses. Aphids originated in the late Cretaceous about 100 million years ago, but the Aphidinae which comprises about half of the 4700 described species and genera of aphids alive today come from their most recent radiation which occurred in the late Tertiary less than ten million years ago [74], [75]. The aphidines are unusual among phytophagous insects in that many alternate between woody winter hosts, and herbaceous summer host species on which they reproduce parthenogenetically [75]. Aphids locate their preferred hosts by flying from plant to plant, briefly probing all the plants on which they alight along the way until they find those hosts. This behaviour is one of the factors that enables them to be efficient vectors of viruses, such as potyviruses. Thus it is likely that agricultural crops provide aphidines with a predictable succession of suitable summer herbaceous hosts on which they can reproduce rapidly and produce very large migrant populations. Potyviruses have been shown experimentally to adapt to novel host species when serially passaged in them [76], [77], and thus large populations of aphids, repeatedly passaging potyviruses as they migrate through genetically uniform crops, probably provide the conditions that specifically drive potyvirus speciation. Since the mid-Holocene, agriculture has spread around the world, on foot at first, enabling aphids and potyviruses to spread, speciate and attain their current dominance.

The major radiation revealed by the phylogeny of extant potyviruses may not necessarily have coincided with the event that produced the first potyvirus, for while the progenitor potyvirus was constrained as a single population, recombination and successive selective ‘sweeps’ within that population would have ensured that its origin could not be revealed by phylogenetic analysis. However when, as a result of host specialization and geographical separation, discrete lineages were able to persist as separate diverging populations, then evidence of the divergence could be detected by comparing gene sequences from the separate populations.

Diamond [41] has linked the emergence and spread of human ‘crowd diseases’, such as ‘flu, measles and smallpox, with the rise of agriculture. He suggested that increases in the populations of human beings and the animals they domesticated, and the close interaction between those populations, led to the emergence of zoonotic diseases and their eventual specialization and restriction to the human population. In this paper we have reported our conclusion that, for basically the same reasons, agriculture also stimulated the emergence of the potyviruses.and their dominance in crops.

## Supporting Information

List S1 (0.03 MB DOC)

List S2 (0.03 MB DOC)

List S3 (0.03 MB DOC)

List S4 (0.02 MB DOC)

List S5 (0.03 MB DOC)

List S6 (0.02 MB DOC)

List S7 (0.03 MB DOC)
