# Supplementary material for: The Prehistory of Potyviruses: Their Initial Radiation Was during the Dawn of Agriculture
Source: PLoS One. 2008 Jun 25;3(6):e2523. doi: 10.1371/journal.pone.0002523 (PMC2429970; doi:10.1371/journal.pone.0002523)
Supplement: List S1 — (0.03 MB DOC) [file pone.0002523.s001.doc]

**Supporting Information List 1.**

**Accession Codes of the sequences of the ‘outgroup potyviruses’ and ryegrass mosaic virus;** Bean common mosaic virus AJ312437; Bean common necrotic mosaic virus AY138897; Bean yellow mosaic virus D28819; Beet mosaic virus AY206394; Chilli veinal mottle virus AJ237843; Clover yellow vein virus [AB011819](http://www.ncbi.nlm.nih.gov/entrez/query.fcgi?cmd=Retrieve&db=Nucleotide&list_uids=2960543&dopt=GenBank); Cocksfoot streak virus [AF499738](http://www.ncbi.nlm.nih.gov/entrez/query.fcgi?cmd=Retrieve&db=Nucleotide&list_uids=20336645&dopt=GenBank); Cowpea aphid-borne mosaic virus [AF348210](http://www.ncbi.nlm.nih.gov/entrez/query.fcgi?cmd=Retrieve&db=Nucleotide&list_uids=19070508&dopt=GenBank); Daphne virus Y [DQ299908](http://www.ncbi.nlm.nih.gov/entrez/query.fcgi?cmd=Retrieve&db=Nucleotide&list_uids=83596450&dopt=GenBank); Dasheen mosaic virus [AJ298033](http://www.ncbi.nlm.nih.gov/entrez/query.fcgi?cmd=Retrieve&db=Nucleotide&list_uids=16075312&dopt=GenBank); East Asian Passiflora virus [AB246773](http://www.ncbi.nlm.nih.gov/entrez/query.fcgi?cmd=Retrieve&db=Nucleotide&list_uids=85539887&dopt=GenBank); Fritillary virus Y AM039800; Japanese yam mosaic virus AB027007; Johnsongrass mosaic virus [Z26920](http://www.ncbi.nlm.nih.gov/entrez/query.fcgi?cmd=Retrieve&db=Nucleotide&list_uids=2624186&dopt=GenBank); Konjak mosaic virus [AB219545](http://www.ncbi.nlm.nih.gov/entrez/query.fcgi?cmd=Retrieve&db=Nucleotide&list_uids=89941438&dopt=GenBank); Leek yellow stripe virus [AJ307057](http://www.ncbi.nlm.nih.gov/entrez/query.fcgi?cmd=Retrieve&db=Nucleotide&list_uids=18564832&dopt=GenBank); Lettuce mosaic virus [X97705](http://www.ncbi.nlm.nih.gov/entrez/query.fcgi?cmd=Retrieve&db=Nucleotide&list_uids=1903232&dopt=GenBank); Lily mottle virus [AJ564636](http://www.ncbi.nlm.nih.gov/entrez/query.fcgi?cmd=Retrieve&db=Nucleotide&list_uids=38707330&dopt=GenBank); Maize dwarf mosaic virus [AJ001691](http://www.ncbi.nlm.nih.gov/entrez/query.fcgi?cmd=Retrieve&db=Nucleotide&list_uids=3123635&dopt=GenBank); Narcissus yellow stripe virus [AJ311372](http://www.ncbi.nlm.nih.gov/entrez/query.fcgi?cmd=Retrieve&db=Nucleotide&list_uids=28301538&dopt=GenBank); Onion yellow dwarf virus [AJ510223](http://www.ncbi.nlm.nih.gov/entrez/query.fcgi?cmd=Retrieve&db=Nucleotide&list_uids=31414243&dopt=GenBank); Papaya leaf distortion mosaic virus AB088221; Papaya ringspot virus [X67673](http://www.ncbi.nlm.nih.gov/entrez/query.fcgi?cmd=Retrieve&db=Nucleotide&list_uids=61350&dopt=GenBank); Pea seed-borne mosaic virus [D10930](http://www.ncbi.nlm.nih.gov/entrez/query.fcgi?cmd=Retrieve&db=Nucleotide&list_uids=220995&dopt=GenBank); Peanut mottle virus [AF023848](http://www.ncbi.nlm.nih.gov/entrez/query.fcgi?cmd=Retrieve&db=Nucleotide&list_uids=2739029&dopt=GenBank); Peanut stripe virus U05771; Pennisetum mosaic virus [AY642590](http://www.ncbi.nlm.nih.gov/entrez/query.fcgi?cmd=Retrieve&db=Nucleotide&list_uids=55247451&dopt=GenBank); Pepper mottle virus [M96425](http://www.ncbi.nlm.nih.gov/entrez/query.fcgi?cmd=Retrieve&db=Nucleotide&list_uids=332869&dopt=GenBank); Peru tomato mosaic virus [AJ437280](http://www.ncbi.nlm.nih.gov/entrez/query.fcgi?cmd=Retrieve&db=Nucleotide&list_uids=28460475&dopt=GenBank); Plum pox virus virus [D13751](http://www.ncbi.nlm.nih.gov/entrez/query.fcgi?cmd=Retrieve&db=Nucleotide&list_uids=222408&dopt=GenBank); Potato virus A Z21670; Potato virus V AJ243766; Potato virus Y X12456; Scallion mosaic virus [AJ316084](http://www.ncbi.nlm.nih.gov/entrez/query.fcgi?cmd=Retrieve&db=Nucleotide&list_uids=18621212&dopt=GenBank); Shallot yellow stripe virus AJ865076; Soybean mosaic virus [D00507](http://www.ncbi.nlm.nih.gov/entrez/query.fcgi?cmd=Retrieve&db=Nucleotide&list_uids=11995002&dopt=GenBank); Sugarcane mosaic virus [AJ297628](http://www.ncbi.nlm.nih.gov/entrez/query.fcgi?cmd=Retrieve&db=Nucleotide&list_uids=18621142&dopt=GenBank); Sweet potato feathery mottle virus [D86371](http://www.ncbi.nlm.nih.gov/entrez/query.fcgi?cmd=Retrieve&db=Nucleotide&list_uids=2522084&dopt=GenBank); Thunberg fritillary virus AJ851866; Tobacco etch virus [M15239](http://www.ncbi.nlm.nih.gov/entrez/query.fcgi?cmd=Retrieve&db=Nucleotide&list_uids=335201&dopt=GenBank); Tobacco vein mottling virus [X04083](http://www.ncbi.nlm.nih.gov/entrez/query.fcgi?cmd=Retrieve&db=Nucleotide&list_uids=2598609&dopt=GenBank); Turnip mosaic virus [AF169561](http://www.ncbi.nlm.nih.gov/entrez/query.fcgi?cmd=Retrieve&db=Nucleotide&list_uids=56160434&dopt=GenBank); Watermelon mosaic virus AY437609; Wild potato mosaic virus [AJ437279](http://www.ncbi.nlm.nih.gov/entrez/query.fcgi?cmd=Retrieve&db=Nucleotide&list_uids=25045793&dopt=GenBank); Wisteria vein mosaic virus AY656816; Yam mosaic virus [U42596](http://www.ncbi.nlm.nih.gov/entrez/query.fcgi?cmd=Retrieve&db=Nucleotide&list_uids=1552411&dopt=GenBank); Zucchini yellow mosaic virus [AF127929](http://www.ncbi.nlm.nih.gov/entrez/query.fcgi?cmd=Retrieve&db=Nucleotide&list_uids=17019510&dopt=GenBank); Ryegrass mosaic rymovirus Y09854.
