# Supplementary material for: The Prehistory of Potyviruses: Their Initial Radiation Was during the Dawn of Agriculture
Source: PLoS One. 2008 Jun 25;3(6):e2523. doi: 10.1371/journal.pone.0002523 (PMC2429970; doi:10.1371/journal.pone.0002523)
Supplement: List S2 — (0.03 MB DOC) [file pone.0002523.s002.doc]

**Supporting Information List 2.**

**Accession Codes of the sequences of the representative cCP sequences used to test evolutionary methods and models:** A. cowpea aphid-borne mosaic virus sequences: AF083558, AF241233, AF348210, AF368424, AJ132414, AY253906, AY253907, AY253908, AY253909, AY253910, AY253911, AY433950, AY433951, AY433952, AY434454, AY505342, D10053, DQ397527, DQ397528, DQ397529, DQ397530, DQ397531, DQ397532, S51666, U90326, X82873, Y17822, Y17824, Y18634. B. papaya ringspot virus sequences: AB127935 AF063220, AF319484, AF319493, AF319497, AF506845, AF506857, AF506860, AF506862, AF506867, AF506868, AF506885, AJ012649, AJ012650, AR908084, AY491011, AY839865, DD104495, DQ085863, DQ088670, DQ104819, DQ339580, DQ340769, DQ354071, DQ374152, DQ374153, DQ419573, DQ666640, EF210196, EF210197
