# Supplementary material for: The Prehistory of Potyviruses: Their Initial Radiation Was during the Dawn of Agriculture
Source: PLoS One. 2008 Jun 25;3(6):e2523. doi: 10.1371/journal.pone.0002523 (PMC2429970; doi:10.1371/journal.pone.0002523)
Supplement: List S3 — (0.03 MB DOC) [file pone.0002523.s003.doc]

**Supporting Information List 3.**

**Accession Codes of the sequences of 227 potyvirid sequences.** A08776, AB011819, AB027007, AB079886, AB079887, AB100443, AB126033, AB188115, AB194787, AB194792, AB194797, AB218280, AB219545, AB219833, AF014811, AF023848, AF035818, AF169561, AF189125, AF228516, AF237963, AF285169, AF285170, AF363639, AF394601, AF394602, AF401295, AF401296, AF454454, AF454455, AF501591, AF522296, AF530055, AF536942, AF543212, AF543709, AJ131400, AJ131401, AJ131402, AJ131403, AJ242725, AJ243957, AJ252242, AJ278405, AJ297628, AJ308472, AJ308473, AJ308474, AJ308475, AJ308476, AJ308477, AJ312437, AJ312438, AJ312439, AJ312774, AJ316084, AJ437280, AJ544266, AJ544267, AJ851866, AJ885005, AJ889866, AM039800, AM048875, AM113988, AM157175, AM181350, AM182028, AY010722, AY027810, AY028309, AY042184, AY090660, AY112735, AY134473, AY149118, AY162218, AY166866, AY166867, AY184478, AY188994, AY192568, AY206394, AY216987, AY231130, AY278998, AY278999, AY279000, AY282577, AY294045, AY569692, AY575773, AY609385, AY609386, AY623626, AY623627, AY626825, AY745491, AY745492, AY823985, AY823986, AY833735, AY884982, AY884983, AY884984, AY884985, AY912055, AY912056, AY912057, AY912058, AY953262, AY953264, AY953266, AY968604, AY994084, D00441, D00507, D01091, D10930, D13751, D83408, D86371, DQ124239, DQ299908, DQ309028, DQ340769, DQ340770, DQ340771, DQ345522, DQ374152, DQ374153, DQ399708, DQ450199, DQ465242, DQ465243, DQ648591, DQ648592, DQ666332, DQ674263, DQ674264, DQ821938, DQ821939, DQ851493, DQ851494, DQ851495, DQ851496, DQ977725, DQ986288, EF016294, EF017707, EF026074, EF026075, EF026076, EF062582, EF183499, EF374098, EF558545, L49381, M15239, M92280, M96425, NC_000947, NC_001445, NC_001517, NC_001555, NC_001616, NC_001785, NC_001814, NC_001841, NC_001886, NC_002350, NC_002509, NC_002990, NC_003224, NC_003377, NC_003398, NC_003483, NC_003492, NC_003501, NC_003537, NC_003605, NC_003606, NC_003742, NC_003797, NC_004010, NC_004011, NC_004013, NC_004016, NC_004035, NC_004039, NC_004047, NC_004426, NC_004752, NC_005028, NC_005029, NC_005136, NC_005288, NC_005778, NC_005904, NC_006262, NC_006941, NC_007147, NC_007180, NC_007216, NC_007433, NC_007728, NC_008028, NC_008558, NC_009741, NC_009742, NC_009743, NC_009744, NC_009745, S42280, U09509, U34972, U47033, U57358, U58770, X16415, X67673, X69757, X73883, X89997, X97251, X97704, Z21670,
