# Supplementary material for: The Prehistory of Potyviruses: Their Initial Radiation Was during the Dawn of Agriculture
Source: PLoS One. 2008 Jun 25;3(6):e2523. doi: 10.1371/journal.pone.0002523 (PMC2429970; doi:10.1371/journal.pone.0002523)
Supplement: List S4 — (0.02 MB DOC) [file pone.0002523.s004.doc]

**Supporting Information List 4.**

**Accession Codes of the sequences of 67 PPV sequences.** AF172346, AF172347, AF172349, AF332871, AF360579, AF401295, AF401296, AF440741, AF440742, AF440743, AF440744, AF440745, AF440746, AJ000340, AJ243957, AJ306420, AJ566344, AJ566345, AJ566346, AM157175, AM184114, AM260933, AM260934, AM260935, AM260936, AM260937, AY028309, AY591253, AY591254, AY677115, AY690605, AY690609,

AY912055, AY912056, AY912057, AY912058, AY953261, AY953264, AY953267, D13751, DQ299537, DQ299538, DQ423227, DQ423228, DQ423230, DQ423231, DQ423234, DQ423238, DQ423239, DQ465243, DQ883816, M21847, M92280, S57404, S57405, X16415, X56258, X57975, X57976, X81073, X81074, X81075, X81076, X81077, X81078, X81079, X81080, X81081, X81082, X81083, X97398
