# Supplementary material for: The Prehistory of Potyviruses: Their Initial Radiation Was during the Dawn of Agriculture
Source: PLoS One. 2008 Jun 25;3(6):e2523. doi: 10.1371/journal.pone.0002523 (PMC2429970; doi:10.1371/journal.pone.0002523)
Supplement: List S5 — (0.03 MB DOC) [file pone.0002523.s005.doc]

**Supporting Information List 5.**

**Accession Codes of the sequences of 38 Australian potyvirus isolates.** *Apium* virus Y AF207594, AF203529 and AY049716; Bean common mosaic virus AY850005; Bean yellow mosaic AB041972, AF042272, AF185960/62, AF192781-3, AF434661, AJ844916, AY376314, AY397612, DQ901431-5, S77515, U47033, U78191, X81124 and X53684; Carrot virus Y AF203537-9; Celery mosaic virus AF203532-5; Ceratobium mosaic virus AF022442-6; *Clitoria* virus Y AF228515; Clover yellow vein virus AF203536, AF185959, S77521 and AY169801; *Dianella* chlorotic mottle virus DQ075247; *Diuris* virus Y AF203527; *Euphorbia* ringspot virus AY517544; *Eustrephus* virus Y DQ098904; *Glycine* virus Y DQ098902; *Hardenbergia* mosaic virus DQ898188-214, EF375606-8; *Hibbertia* virus Y AF228516; Johnson grass mosaic virus AF032404; AY387806-28; X05040; Z26920; *Kennedya* virus Y DQ098903; *Ornithogalum* mosaic virus AF203528; Papaya ringspot virus U14736-40; U14744; *Passiflora foetida* virus Y DQ112219; Passion fruit woodiness virus AJ430527; DQ898215-18 and U67149-51; Pea seed-borne mosaic virus AF127767; Peanut mottle virus X73422; *Pleione* virus Y AF185958 ; Potato virus Y Brendan Rodoni, personal communication; *Pterostylis* virus Y AF185964-5 ; *Rhopalanthe* virus Y AF185956 ; *Sarcochilus* virus Y AF185956; Siratro 1 virus Y DQ098900; Siratro 2 virus Y DQ098901; Sugarcane mosaic virus AF006728 – 38, AJ278405 and D00948; Sweet potato feathery mottle virus-C AJ781778-9, AM050891-3; Sweet potato feathery mottle virus–RC AJ781775-7, AM050889-90; Sweet potato virus-Y AM050884-88; Turnip mosaic virus AB076543, AF228514, AF226846; Watermelon mosaic virus D00535; Zucchini yellow mosaic virus S81377, S81381, S81384, S81387.
