# Supplementary material for: The Prehistory of Potyviruses: Their Initial Radiation Was during the Dawn of Agriculture
Source: PLoS One. 2008 Jun 25;3(6):e2523. doi: 10.1371/journal.pone.0002523 (PMC2429970; doi:10.1371/journal.pone.0002523)
Supplement: List S6 — (0.02 MB DOC) [file pone.0002523.s006.doc]

**Supporting Information List 6.**

**Accession Codes of the sequences of 25 CAbMV sequences.** AF083558, AF241233, AF368424, AJ132414, AY253906, AY253908, AY253910, AY253911, AY433950, AY433951, AY433952, AY434454, AY505342, D10053, DQ397527, DQ397529, DQ397530, DQ397532, EF547367, NC_004013, S51666, U90326, X82873, Y17822, Y17824
