# Supplementary material for: The Prehistory of Potyviruses: Their Initial Radiation Was during the Dawn of Agriculture
Source: PLoS One. 2008 Jun 25;3(6):e2523. doi: 10.1371/journal.pone.0002523 (PMC2429970; doi:10.1371/journal.pone.0002523)
Supplement: List S7 — (0.03 MB DOC) [file pone.0002523.s007.doc]

**Supporting Information List 7.**

**Accession Codes of the sequences of 327 PRSV sequences.** AB044339, AB044340, AB044341, AB044342, AB127935, AF063220, AF063221, AF120270, AF196838, AF196839, AF243496, AF307778, AF309968, AF319482, AF319483, AF319484, AF319485, AF319486, AF319487, AF319488, AF319489, AF319490, AF319491, AF319492, AF319493, AF319494, AF319495, AF319496, AF319497, AF319498, AF319499, AF319500, AF319501, AF319502, AF319503, AF319504, AF319505, AF319506, AF319507, AF344639, AF344640, AF344641, AF344642, AF344643, AF344644, AF344645, AF344646, AF344647, AF344648, AF344649, AF344650, AF374862, AF374863, AF374864, AF374865, AF469065, AF469066, AF506840, AF506841, AF506842, AF506843, AF506844, AF506845, AF506846, AF506847, AF506848, AF506849, AF506850, AF506851, AF506852, AF506853, AF506854, AF506855, AF506856, AF506857, AF506858, AF506859, AF506860, AF506861, AF506862, AF506863, AF506864, AF506865, AF506866, AF506867, AF506868, AF506869, AF506870, AF506871, AF506872, AF506873, AF506874, AF506875, AF506876, AF506877, AF506878, AF506879, AF506880, AF506881, AF506882, AF506883, AF506884, AF506885, AF506886, AF506887, AF506888, AF506889, AF506890, AF506891, AF506892, AF506893, AF506894, AF506895, AF506896, AF506897, AF506898, AF506899, AF506900, AF506901, AF506902, AF506903, AF506904, AF530088, AF530089, AJ012099, AJ012649, AJ012650, AJ875101, AJ875102, AJ875103, AJ875104, AJ875105, AJ875106, AJ875107, AJ875108, AJ875109, AJ875110, AJ875111, AJ875112, AJ875113, AJ875114, AJ875115, AJ875116, AR095131, AR095132, AR095136, AR095137, AR095138, AR095139, AR095140, AR095141, AR908078, AR908079, AR908080, AR908081, AR908082, AR908083, AR908084, AR908085, AR908086, AR908087, AY010712, AY010713, AY010714, AY010715, AY010716, AY010717, AY010718, AY010719, AY010720, AY010721, AY010722, AY017189, AY017190, AY027810, AY027811, AY027812, AY094984, AY094985, AY094986, AY094987, AY162218, AY231130, AY238880, AY238881, AY238882, AY238883, AY238884, AY238885, AY423557, AY458617, AY458618, AY458619, AY458620, AY491011, AY587583, AY687386, AY839863, AY839864, AY839865, AY841757, AY903266, D00594, D00595, D50591, DD104494, DD104495, DD104496, DD104497, DD104498, DD104499, DD104500, DD104501, DD104502, DD104503, DQ008446, DQ008447, DQ008448, DQ008449, DQ077175, DQ085856, DQ085857, DQ085858, DQ085859, DQ085860, DQ085861, DQ085862, DQ085863, DQ085864, DQ085865, DQ085866, DQ088670, DQ088671, DQ089482, DQ104812, DQ104813, DQ104814, DQ104815, DQ104816, DQ104817, DQ104818, DQ104819, DQ104820, DQ104821, DQ104822, DQ104823, DQ192587, DQ339576, DQ339577, DQ339578, DQ339579, DQ339580, DQ339581, DQ340769, DQ340770, DQ340771, DQ354071, DQ354072, DQ374152, DQ374153, DQ419573, DQ449532, DQ449533, DQ449534, DQ449535, DQ449536, DQ500093, DQ500094, DQ500095, DQ500096, DQ500097, DQ500098, DQ500099, DQ500100, DQ500101, DQ500102, DQ500103, DQ500104, DQ500105, DQ500106, DQ500107, DQ500108, DQ500109, DQ500110, DQ500111, DQ500112, DQ500113, DQ500114, DQ500115, DQ500116, DQ500117, DQ641510, DQ641511, DQ650651, DQ666638, DQ666639, DQ666640, DQ666641, DQ868880, E12704, EF017707, EF104919, EF183499, EF210196, EF210197, NC_001785, S46722, S89893, U14736, U14736, U14737, U14737, U14738, U14738, U14739, U14739, U14740, U14740, U14741, U14742, U14743, U14744, X67672, X67673, X78557, X96538, X97251
